# ﻿Karyotype and COI gene sequences of *Chironomusmelanotus* Keyl, 1961 from the Yaroslavl region, Russia, and the difficulties with its identification using GenBank and BOLD systems

**DOI:** 10.3897/compcytogen.v16.i3.90336

**Published:** 2022-09-14

**Authors:** Viktor V. Bolshakov, Ekaterina A. Movergoz

**Affiliations:** 1 Papanin Institute for Biology of Inland Waters Russian Academy of Sciences, Yaroslavl reg., Nekouz prov., Borok, 152742, Russia Papanin Institute for Biology of Inland Waters Russian Academy of Sciences Borok Russia

**Keywords:** Chironomidae, *Chironomusmelanotus*, *COI*, dark taxa, DNA-barcode, karyotype

## Abstract

Karyotype and *COI* gene sequences of *Chironomusmelanotus* Keyl, 1961 from the Yaroslavl region (Russia) were analyzed. A low level of chromosomal polymorphism has been confirmed, eventually eight banding sequences were found: melA1, melB1, melC1, melD1, melE1, melF1, and melG1; only melD2 was found in two larvae from the Sunoga river. Analysis of phylogenetic tree and estimated genetic distances has shown not all *COI* gene sequences of *Ch.melanotus* in GenBank and BOLD to belong to this species. The lower distance of 0.4% was observed between two sequences from the Yaroslavl region and Finland, apparently these are true *Ch.melanotus* sequences. The distances between true *Ch.melanotus* and other sequences from Finland were 9.5% and 12.4%, and from Sweden it was 11%. The average genetic distance between studied sequences of 9.1% is out of the range of the 3% threshold previously determined for chironomids. According to our estimates, there are two sequences with a distance of 2.9% that may belong to *Ch.annularius* Meigen, 1818, and one sequence with a genetic distance of 2.1%, may belonging to *Ch.cingulatus* Meigen, 1830, which has been confirmed karyologically. Another two sequences form a separate cluster. We suggest that they either belong to a known species, but are not present in the databases, or belong to a distinct, undescribed species.

## ﻿Introduction

*Chironomusmelanotus* Keyl, 1961 is one of the most widespread and well-known species. It does not belong to any sibling species group (Kiknadze et al. 2010). The first finding and description was in Germany (Keyl 1961, 1962; Degelmann et al. 1979). *Ch.melanotus* is in demand in classical hydrobiology (Fjellheim, Raddum 1996) and in toxicology (Grebenjuk 1994; Grebenjuk and Tomilina 2014). The main problem in the investigation of *Chironomus* Meigen, 1803 is the difficulties with the species identification by larval morphology. Due to the presence of the giant chromosomes in the salivary gland of *Chironomus* larvae, it is more convenient to identify cytogenetically (Kiknadze et al. 1991, 2016). The karyotype of *Ch.melanotus* was described by Keyl (1961) as a “cytospecies” that belongs to “thummi” cytocomplex and mapped chromosomal arms A and F (Keyl, 1962). It has been shown that the level of polymorphism in *Ch.melanotus* is very low (Wülker 1973; Kiknadze et al. 1991; Jabłońska-Barna et al. 2013). Only in polluted water bodies a high spectrum of somatic rearrangements and a case of trisomy were observed (Jabłońska-Barna et al. 2013). Finally, due to the development of new techniques in molecular biology, for species identification/delimitation the fast and cost-effective technology DNA barcoding is commonly used and for massive analysis in biomonitoring metabarcoding is used. In recent years, many works on this theme have been published. This is a barcoding of invertebrates, including chironomids from Canada (Hebert et al. 2016), Germany (Morinière et al. 2019), Finland (Roslin et al. 2022), South Korea (Kang et al. 2022), Montenegro/Albania (Gadawski et al. 2022) and others. The disadvantage of this approach is the presence in the databases of genetic information (GenBank and BOLD) from unidentified or incorrectly identified specimens, so-called “dark taxa” (Morinière et al. 2019). The next problem is the understanding that a sequence divergence threshold is not suitable for all *Chironomus* species and depends on intraspecific and interspecific sequence divergences. Interspecific - varied for *COI* gene sequences in most cases from 9 to 20% and in rare cases from 1 to 4% (Proulx et al. 2013). Due to the fact that we cannot fully estimate intra- and inter-specific sequence divergences, here we will use the average value of this parameter – 3% (Ekrem et al. 2007; Proulx et al. 2013; Kondo et al. 2016).

We could not find any studies of *Ch.melanotus* involving approaches of morphology, cytogenetics and DNA barcoding published in one article. In the GenBank and BOLD databases were found five and one *COI* gene sequences, respectively. These sequences were obtained from individuals collected in Finland and Sweden, and deposited during the preparation of this paper (Roslin et al. 2022). Preliminary examination has shown that not all of these sequences belong to *Ch.melanotus*.

The present study aims to calculate and compare the genetic distances between *COI* gene sequences of *Ch.melanotus* from Yaroslavl region identified by morphology and cytogenetics and the sequences obtained from GenBank and BOLD of *Ch.melanotus* from different populations identified by morphology or molecular-genetics (barcode), and additional sequences from GenBank and BOLD of several *Chironomus* identified by cytogenetics.

## ﻿Materials and methods

Fourth instar larvae of *Ch.melanotus* were collected from a few places in the Yaroslavl region, Russia. Thirty-one larvae were found in a puddle on the Shumarovka river shore (58°02'25.5"N, 38°15'33.2"E) in October 2018. The depth is 0.5 m, and the bottom is black silt. Seven larvae were collected in the Sunoga river (58°03'20.3"N, 38°14'04.2"E) in August 2018. The depth is 0.1 – 0.2 m, and the bottom is gray silt with sand. Four larvae were collected in a small stream (brook) in the shore zone of the Kotorosl’ river (57°22'41.6"N, 39°50'08.5"E) in June 2016. The depth is 0.5 m, and the bottom is black silt and rotting wood.

The age was determined by the standard method (Ilyinskaya, 1983). All larvae were taken for karyotype analysis using the ethanol-orcein technique (Dyomin 1989). A Micromed-6C (LOMO, St. Petersburg) light microscope equipped with a standard (kit) oil objective ×100 and a camera ToupCam5.1 (China) were used for microscopy analysis. To identify chromosome banding sequences, the cytomaps by Kiknadze et al. (1991, 2016), Keyl (1961, 1962), Hirvenoja and Michailova (1991) were used. Preparations of *Ch.melanotus* have been deposited at IBIW RAS.

One larva from a small stream (brook) in the shore zone of Kotorosl river studied karyologically was taken for the total DNA extraction using “M-sorb-OOM” (Sintol, Moscow) kit with magnet particles according to manufacturer’s protocol. For amplification of *COI* (cytochrome oxidase subunit I) we used primers LCO1490 (5’-GGTCAACAAATCATAAAGATATTGG-3’) and HCO2198 (5’-TAAACTTCAGGGTGACCAAAAAATCA -3’) (Eurogen, Moscow) (Folmer et al. 1994). Amplification reaction was carried out in 25 μl reaction mixture (1x buffer, 1.5 μМ MgCl2, 0.5 mM of each primer, 0.2 μМ dNTP of each nucleotide, 17.55 μL deionized water, 1 μL template DNA, 1 unit Taq-polymerase (Evrogen, Moscow). PCR was performed at 94 °C (3 min), followed by 30 cycles at 94 °C (15 s), 50 °C (45 s), 72 °C (60 s) and a final extension at 72 °C (8 min). PCR products were visualized on 1% agarose gels and later purified by ethanol and ammonium acetate (3 M). Both strands were sequenced on an Applied Biosystems 3500 DNA sequencer (Thermo Scientific, USA) following the manufacturer’s instructions.

For alignment of *COI* nucleotide sequences, we used MUSCLE in the MEGA6 software (Tamura et al. 2013). The MEGA6 was used to calculate pairwise genetic distances (p-distance) with codon position preferences: 1^st^, 2^nd^, 3^rd^ and noncoding sites. The Bayesian analysis was performed using the program MrBayes v.3.2.6 (Ronquist and Huelsenbeck 2003; Ronquist et al. 2012) with settings suggested by Karmokov (2019; Bolshakov, Prokin 2021), for 1 000 000 iterations and 1000 iterations of burn-in, nst = 6 (GTR + I + G). The phylogenetic trees resulting in Bayesian inference analyses were visualized and edited using FigTree v.1.4.3 (http://tree.bio.ed.ac.uk/software/figtree/).

In addition, thirty-four *COI* gene sequences of the genus *Chironomus* from “GenBank” and “Barcode of Life Data Systems” (BOLD) were analyzed. Accession numbers of used sequences in GenBank and BOLD: *Ch.acutiventris* Wülker et al., 1983 (AF192200.1), *Ch.annularius* Meigen, 1818 (AF192189.1), *Ch.anthracinus* Zetterstedt, 1860 (KF278222), *Ch.balatonicus* Devai et al., 1983 (JN016826.1), *Ch.bernensis* Wülker et Klötzli, 1973 (AF192188.1), *Ch.borokensis* Kerkis et al., 1988 (AB740261), *Ch.cingulatus* Meigen, 1830 (AF192191.1), *Ch.commutatus* Keyl, 1960 (AF192187.1), *Ch.curabilis* et al., 1990 (JN016810.1), *Ch.dilutus* et al., 1999 (KF278335.1), *Ch.entis* Shobanov, 1989 (KM571024.1), *Ch.heterodentatus* Konstantinov, 1956 (AF192199.1), *Ch.heteropilicornis* Wülker, 1996 (MK795770.1), *Ch.maturus* Johannsen, 1908 (DQ648204.1), *Ch.melanotus* (MZ659620, MZ657748, MZ658877, MZ657558, MZ658420, BSCHI737-17), *Ch.nipponensis* Tokunaga, 1940 (DQ648206), *Ch.novosibiricus* Kiknadze et al., 1993 (AF192197.1), *Ch.nuditarsis* Keyl, 1961 (KY225345.1), *Ch.obtusidens* Goetghebuer, 1921 (CHMNO207-15*), *Ch.piger* Strenzke, 1959 (AF192202.1), *Ch.pilicornis* Fabricius, 1787 (HM860166.1), *Ch.plumosus* Linnaeus, 1758 (KF278217.1), *Ch.riparius* Meigen, 1804 (KR756187.1), *Ch.sokolovae* Istomina et al., 1999 (MW471100), *Ch.sororius* Wulker, 1973 (MZ324811), *Ch.tentans* Fabricius, 1805 (AF110157.1), *Ch.tuvanicus* Kiknadze et al., 1993 (AF192196.1), *Ch.usenicus* Loginova et Belyanina, 1994 (JN016820.1), *Ch.whitseli* Sublette et Sublette, 1974 (KR683438.1). The *COI* sequence of *Drosophilamelanogaster* Meigen, 1830 (HQ551913) was used as outgroup in phylogenetic analysis.

## ﻿Results and discussion

### ﻿The karyotype of *Chironomusmelanotus* Keyl, 1961 from the Yaroslavl region, Russia

**Figure 1.**
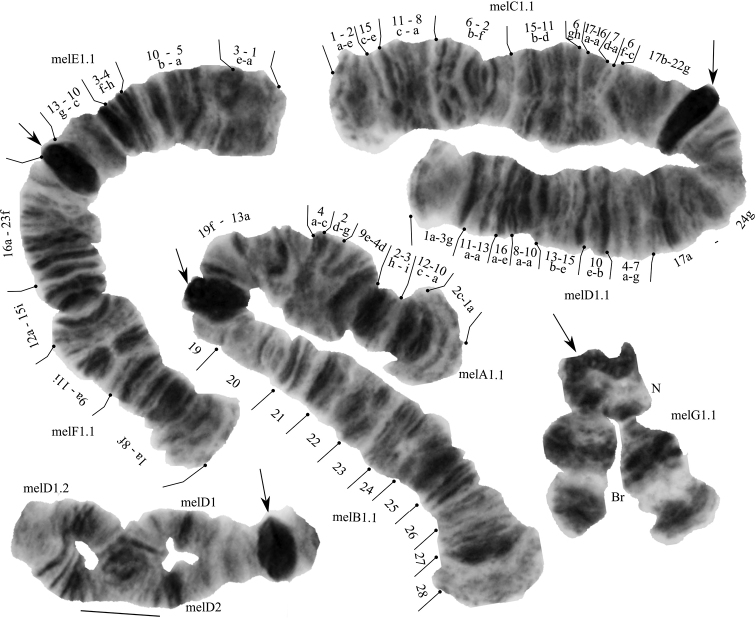
Karyotype of *Chironomusmelanotus* from the Sunoga river, Yaroslavl, Russia. Arrows indicate centromeric bands, melA1, melB1 and etc. – genotypic combinations of banding sequences in chromosome arms, BR – Balbiani rings, N – nucleous.

The species has a 2n = 8 set of chromosomes. By the chromosome arm combination – AB, CD, EF and G, the species belongs to *Chironomus* “thummi” cytocomplex. The chromosomes AB and CD are metacentric, EF is submetacentric, and G is telocentric. The nucleus and Balbiani ring were found in arm G. The peculiarity of the karyotype of *Ch.melanotus* is a heterochromatinized centromeric region that forms an unstable chromocenter (Fig. 1), also observed only in *Ch.cucini* Webb, 1969, *Ch.pilicornis*, *Ch.athalassicus* Cannings 1975, *Ch.magnus* White et Ramsey, 2015 and *Ch.hyperboreus* Staeger, 1845 (Wülker and Butler 1983; Int Panis et al. 1994; Kiknadze and Istomina 2000; Wülker and Martin 2000; Kiknadze et al. 2010).

We found two zygotic combinations: melA1.1. B1.1. C1.1. D1.1. E1.1. F1.1. G1.1, and melA1.1. B1.1. C1.1. D1.2. E1.1. F1.1. G1.1, which was found only in two larvae from the Sunoga river.

All eight banding sequences coincide with banding sequences in Keyl et al. (1961, 1962), Hirvenoja and Michailova (1991) and Kiknadze et al. (1991, 2016).

**Arm A**. One banding sequence: melA1 1a-2c 10a-12c 3i-2h 4d-9e 2g-d 4c-a 13a-19f C.

**Arm B.** One banding sequence: melB1 28-27-26-25-24-23-22-21-20-19 C (mapped according to Hirvenoja, Michailova 1991). Different from *Ch.plumosus* by four inversion steps.

**Arm C.** One banding sequence: melC1 1a-2e 15c-e 11c-8a 6b-2f 15b-11d 6gh 17a-16a 7d-a 6f-c 17b-22g C.

**Arm D.** Two banding sequences: melD1 1a-3g 11a-13a 16a-e 8a-10a 13b-15e 10e-b 4a-7g 17a-24g C in heterozygous state with melD2 1a-3g 11a-13a 16a-e 8a-9e 7g-4a 10b-e 15e-13b 10a 17a-24g C.

**Arm E.** One banding sequence: melE1 1a-3e 5a-10b 4h-3f 10c-13g C.

**Arm F.** One banding sequence: melF1 1a-8f 9a-11i 12a-15i 16a-23f C.

**Arm G.** One banding sequence: melG1. Not mapped.

The level of polymorphism in *Ch.melanotus* is known to be very low (Kiknadze et al. 1991; Jabłońska-Barna et al. 2013). At the moment, we know of two alternative banding sequences, melB2 and melD2, and the sequence melD2 is more typical for Western Siberia populations (Kiknadze et al. 2016) and a Finnish population (Hirvenoja et Michailova 1991). We found melD2 in a heterozygous state in two larvae from the Sunoga river. Any deviations in the karyotype characteristics like a trisomy, rearrangements, insertions and deletions, more typical for polluted water bodies (Jabłońska-Barna et al. 2013) were not found.

### ﻿DNA-barcoding and phylogenetic analysis

The obtained *COI* gene sequence for *Ch.melanotus* from the Yaroslavl region was deposited in the GenBank with accession number OL546775; the length of the sequence is 658 bp (percentage of nucleotides A: 25; T: 38; G: 17; C: 19).

More interesting was the analysis of *COI* gene sequences. As was said previously, for the species name *Ch.melanotus* in the databases match six sequences of the *COI* gene from Finland (MZ659620, MZ657748, MZ658877, MZ657558, MZ658420) identified by molecular-genetics and Sweden (BSCHI737-17) identified by imago characters, and the average genetic distance between them of 9.1% is out of the range of 3% distances previously determined for chironomids (Ekrem et al. 2007; Proulx et al. 2013; Kondo et al. 2016). Low chromosomal variability of *Ch.melanotus* does not allow us to talk about a high level of genetic diversity. We can conclude not all sequences belong to *Ch.melanotus* species (Table 1). According to our estimation, the lower distance, about 0.4%, was between *Ch.melanotus* (MZ659620) from Finland and Yaroslavl reg. (OL546775). The distance between sequences of *Ch.melanotus* (OL546775) from Yaroslavl reg. and sequences from Finland (MZ657748, MZ658877) - 9.5%, (MZ657558, MZ658420) - 12.4%, and Sweden (BSCHI737-17) - 11%. These values are greater than those between sequences from the Yaroslavl reg. (OL546775) and *Ch.anthracinus* (KF278222), identified karyologically (Proulx et al. 2013), with a distance of 4%. This still doesn’t mean these species are really closely related, the analysis of one short segment of the *COI* gene is not enough to make such conclusions (DeSalle et al. 2005). However, a high similarity of their karyotypes has been noted, up to identity of some banding sequences (Keyl 1962; Kiknadze et al. 1991).

**Table 1.** Pairwise genetic distances (p-distances, %) between *COI* gene sequences of *Ch.melanotus* and closest sequences of *Chironomus* from GenBank and BOLD.

	*Ch.melanotus*OL546775 Yaroslavl, RUS	*Ch.melanotus*MZ659620 Finland	*Ch.melanotus*MZ657748 Finland	*Ch.melanotus*MZ658877 Finland	*Ch.melanotus* BSCHI737-17 Sweden	*Ch.melanotus*MZ657558 Finland	*Ch.melanotus*MZ658420 Finland	*Ch.anthracinus* KF278222	*Ch.annularius* AF192189.1	*Ch.cingulatus* AF192191.1
*Ch.melanotus* MZ659620 Finland	0,4									
*Ch.melanotus* MZ657748 Finland	9,5	9,7								
*Ch.melanotus* MZ658877 Finland	9,5	9,7	0,0							
*Ch.melanotus* BSCHI737-17 Sweden	11,0	11,4	7,0	7,0						
*Ch.melanotus* MZ657558 Finland	12,4	12,6	10,7	10,7	11,2					
*Ch.melanotus* MZ658420 Finland	12,4	12,6	10,7	10,7	11,2	0,4				
*Ch.anthracinus* KF278222	4,0	3,6	10,5	10,5	11,6	12,0	12,4			
*Ch.annularius* AF192189.1	12,6	12,8	9,7	9,7	10,1	2,9	2,9	12,6		
*Ch.cingulatus* AF192191.1	10,9	11,2	7,2	7,2	2,1	11,0	11,0	11,8	10,1	
*Ch.nipponensis* DQ648206	10,3	10,7	6,5	6,5	4,2	11,4	11,4	11,2	9,9	4,8

The distance between the two similar sequences (MZ657558 and MZ658420) from Finland and *Ch.annularius* (AF192189.1) confirmed karyologically (Guryev et al. 2001) was 2.9%; between sequences (BSCHI737-17) from Sweden and *Ch.cingulatus* (AF192191.1) confirmed karyologically (Guryev et al. 2001) - 2.1%, *Ch.nipponensis* (DQ648206) - 4.2%, identified by morphology and molecular-genetics (Kondo et al. 2016). Two similar sequences are particularly interesting (MZ657748 and MZ658877), the distances between of them and all the analyzed sequences varied from 6.5 to 10.5%, and the average was 12%. Unfortunately, we didn’t find any matches in GenBank and BOLD.

**Figure 2.**
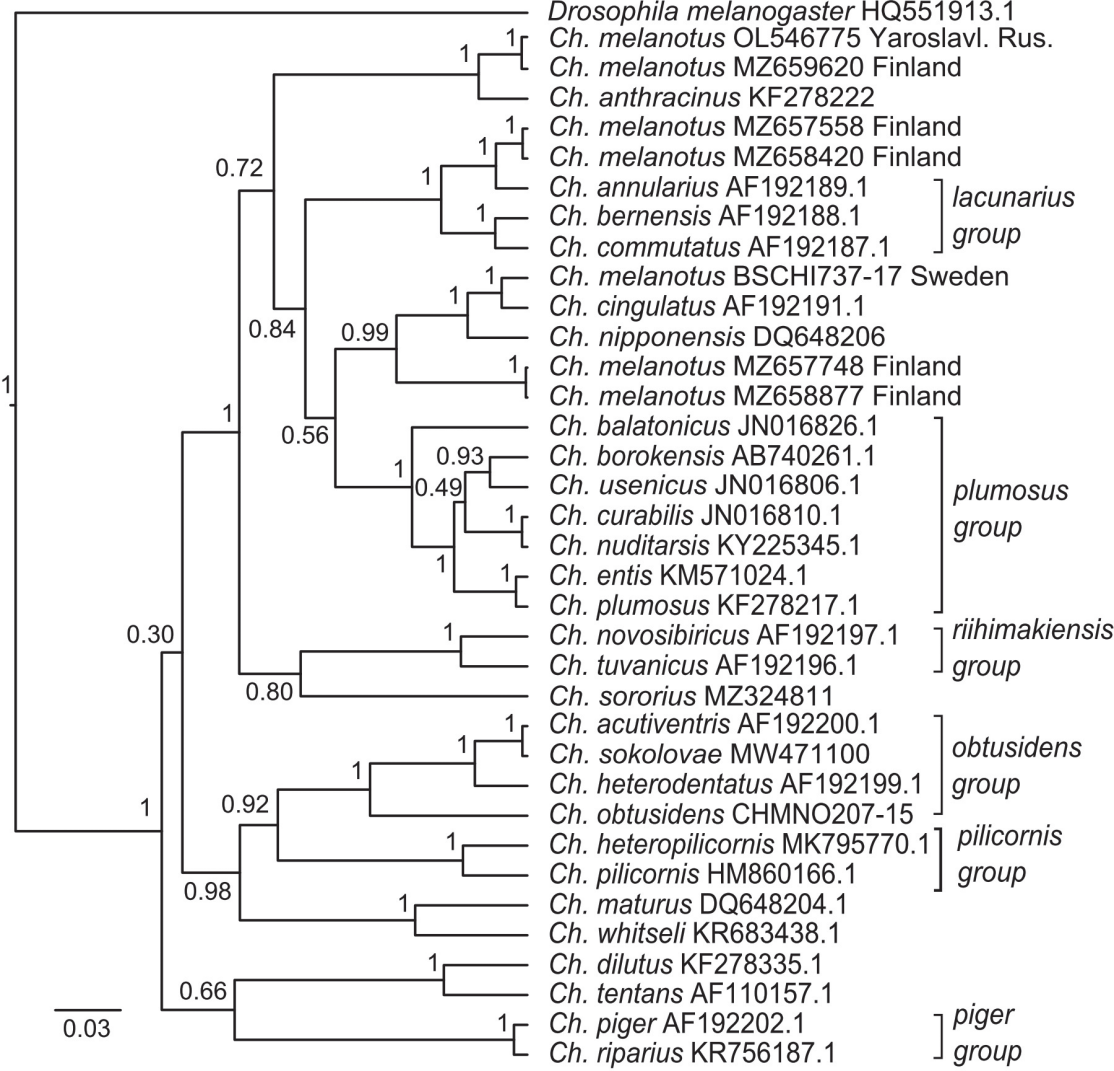
Bayesian tree of the analyzed samples of *Chironomus* spp. inferred from COI sequences. Species name, GenBank accession numbers and group name are shown to the right of the branches. Support values are given if they exceed 0.3. The numbers at the nodes indicate posterior probabilities.

On the phylogenetic tree constructed by Bayesian inference (Fig. 2), we see the same situation as with the genetic distances. The sequence of *Ch.melanotus* (OL546775) from Yaroslavl reg., and *Ch.melanotus* (MZ659620) from Finland combined into one cluster, while the other sequences spread out into different branches. Two similar sequences (MZ657558 and MZ658420) from Finland and *Ch.annularius* (AF192189.1) combined in one cluster, with a support value of 1.0. The sequence (BSCHI737-17) from Sweden and *Ch.cingulatus* (AF192191.1) formed another cluster, with a support value of 1.0. Two similar sequences from Finland (MZ657748 and MZ658877) have formed a separate cluster, without including any other specimens.

All the obtained data show that several species are hidden in GenBank and BOLD under the name “*Chironomusmelanotus*”. First, there is a true *Ch.melanotus* cluster (Fig. 2) (MZ659620 and OL546775) the reliability of which is confirmed by karyotype analysis. Probably, two similar sequences (MZ657558 and MZ658420) belong to *Ch.annularius* (AF192189.1), and the genetic distance of 2.9% is very close to 3% accepted interspecific threshold (Ekrem et al. 2007; Proulx et al. 2013; Kondo et al. 2016), but does not exceed it. Another sequence (BSCHI737-17), with a genetic distance of 2.1%, likely belongs to *Ch.cingulatus* (AF192191.1).

Two similar sequences (MZ657748 and MZ658877) need special attention. The samples of *Ch.melanotus* from Finland were investigated during the project of FinBOL (Finnish Barcode Of Life), in the framework of which the authors tested the system FinPROTAX (Probabilistic Taxonomic Assignment Tool) (Roslin et al. 2022). As the authors report, the accuracy of taxonomic assignments at the level of species reached 88.5% (Roslin et al. 2022). Such precision is still insufficient, especially in a group rich in sibling species. This approach does not consider estimate intra- and inter-specific sequence divergences. For the *COI* gene, the estimated interspecific sequence divergences in most cases varied from 9 to 20%, but in a few cases with well-identified by cytogenetics species, this parameter varied from 1 to 4%, which overlapped between intraspecific and interspecific sequence divergences (Proulx et al. 2013). According to Morinière et al. (2019), the database of genetic information contains about 65% of sequences without species-level assignment, so-called “dark taxa” of all Chironomidae recorded from Germany. But we think that “superficial taxonomic impediment” (species are so poorly and unreliably named, they will need to be redescribed before they can be used) (Meier et al. 2022) is better applicable in this case. Thus, we can conclude that two sequences (MZ657748 and MZ658877) belong to well-known species that are absent in databases, or they can be considered as distinct species. A similar case was with Japanese *Ch.nipponensis*. At first, Yamamoto (2010, cited by Kondo et al. 2016) proposed dividing the “highland” and “lowland” populations of *Ch.nipponensis* by morphology, then Kondo et al. (2016) revealed the genetic distances between them at 9.1%, and confirmed the presence of two separate species.

## ﻿Conclusions

On the example of *Ch.melanotus*, we confirmed that in *Chironomus* species identification we must use all available comprehensive approaches, involving morphological, cytogenetic and molecular genetic studies (mitochondrial and nuclear genes) (DeSalle et al. 2005; Ekrem et al. 2007; Proulx et al. 2013; Kondo et al. 2016).

At least four species of *Chironomus* could be in the databases under the name “*Ch.melanotus*” from Finland and Sweden. This suggests that at the present stage of the collection of genetic data, it is impossible to trust only a computer algorithm. We agree with Zamani et al. (2022) that the use of DNA-based analyses for an initial sorting of new and known species is extremely useful as a first step, which significantly narrows the range of search before precise species identification. Nevertheless, despite the difficulties, the species identification of *Chironomus* greatly enriches our understanding of ecosystem functioning because this is an important part of it.
